# Human antibody heavy chain variable region exon interacts with the 3′ regulatory region to form a stable somatic hypermutation center

**DOI:** 10.1073/pnas.2625638123

**Published:** 2026-09-28

**Authors:** Ying Guan, Mariia Mikhova, Zhuo Yang, Kai Xu, Shuxian Xie, Xin Lin, Adam Yongxin Ye, Taimor Williams, Jianshu Wang, David G. Schatz, Frederick W. Alt, Xi Chen

**Affiliations:** ^a^ Howard Hughes Medical Institute, Boston Children's Hospital, Boston, MA 02115; ^b^ Program in Cellular and Molecular Medicine, Boston Children's Hospital, Boston, MA 02115; ^c^Department of Genetics, Harvard Medical School, Boston, MA 02115; ^d^Department of Immunobiology, Yale School of Medicine, New Haven, CT 06520-8011

**Keywords:** somatic hypermutation, RAMOS, *IGH* 3′RR, chromatin 3D structure, G1-arrest

## Abstract

Activation-induced cytidine deaminase (AID) initiates somatic hypermutation (SHM) of immunoglobulin heavy chain (HC) variable region exons in germinal center (GC) B cells, allowing selection of affinity-matured B cell receptors. The V(D)J exon location is "privileged" for SHM targeting of diverse sequences within it. In analogy to the related HC class switch recombination center, we proposed that cohesin-mediated loop extrusion juxtaposes widely separated enhancers, including 3′ *Igh* regulatory region ("3′RR"), with the V(D)J exon in GC B cells to establish a SHM-center (SHM-C) "privileged" for AID access. To test this hypothesis, we employed a human RAMOS GC B lymphoma cell SHM model with inducible AID expression in which we targeted an unmutated mouse HC V(D)J exon in place of its human counterpart. Activation of AID expression in this cycling RAMOS line for 10 d led to robust SHM accumulation in the mouse V(D)J exon with a pattern similar to that in mouse GC B cells. In this model, the mouse V(D)J exon was indeed juxtaposed to the two human 3′RRs and other elements consistent with SHM-C formation. While deletion of individual 3′RRs had little effect, deletion of both greatly reduced SHM, but left V(D)J exon transcription unabated. Moreover, the SHM-C and SHM accumulation remained intact over a 10-d period in viably G1-arrested and viably G1-arrested RAD21 (cohesin)-depleted versions of the model. We discuss implications for SHM-C structure and function, its long-term functional stability in the absence of the loop extrusion process proposed to assemble it, and for 3′RR SHM functions beyond transcriptional activation.

## Significance

Upon encountering pathogens, B cells enter specialized lymphoid structures known as germinal centers (GCs). GC B cells express activation-induced cytidine deaminase (AID) that initiates somatic hypermutation (SHM) of the portion of antibody genes that encodes antigen recognition. B cells in which mutations increase antibody affinity for activating pathogen are selected to enhance antibody responses. We proposed SHM occurs in a chromosomal structure in the antibody gene locus termed a SHM-center (SHM-C). By studying SHM in a human B cell lymphoma model, we provide direct evidence for existence of a SHM-C assembled from different functional components normally separated by considerable distances. We also find that the SHM-C is surprisingly stable and continues to function for many days in nondividing cells.

The variable region exons of the immunoglobulin heavy chain (*Igh*) locus are assembled in developing B cells through V(D)J recombination. V(D)J recombination generates the primary B cell receptor repertoire (1). Upon exposure to antigen, B cells can undergo two further genomic modifications both initiated by the activation-induced cytidine deaminase (AID) (2). AID initiates *Igh* class switch recombination (CSR) in CSR-activated B cells to exchange expressed *Igh* constant region exons (C_H_s) (3). In germinal center (GC) B cells, AID initiates antigen-induced *Igh* V(D)J exon somatic hypermutation (SHM), allowing affinity maturation of B cell receptors (BCRs) (4, 5). AID initiates both processes by deaminating cytosines within short target motifs that are converted by DNA repair pathways into DNA double-strand breaks (DSBs) for CSR and, predominantly, focal point mutations in V(D)J exons (4, 5). Mechanisms by which AID accesses S regions for CSR are well understood (6, 7), but those by which AID accesses V(D)J exons for SHM are not (5).

In mature mouse B cells, transcription from an *Igh* V(D)J exon runs through C_H_-encoding Cµ, which encodes IgM heavy chains. Upon appropriate antigen or other activation, mature B cells undergo CSR, which replaces Cµ with one of 6 sets of C_H_s 100-200 kb downstream. Mouse C_H_s lie in the order 5′-V(D)J-Cµ-Cδ-Cγ3-Cγ1-Cγ2b-Cγ2a-Cε-Cα-3′ (3). Each C_H_ specifies an antibody class with different pathogen-elimination functions. Long repetitive switch (S) regions precede Cµ and downstream C_H_s (3). AID initiates CSR by deaminating cytosine residues within short sequence target motifs termed RGYW motifs, with the most robust being the palindromic AGCT sequence, that lie within donor Sµ and downstream acceptor S regions (8). Resulting AID-initiated DSBs are repaired through deletional end joining, which ligates the upstream Sµ break to a downstream acceptor S region break, thereby completing CSR (9). The 3′ *Igh* regulatory region ("3′RR") is located at the downstream end of *Igh*, and contains 4 enhancers in a 30 kb region (10). The 3′RR is required for activating acceptor S region transcription, which targets AID, from flanking cytokine/activation-dependent promoters (3, 10–13). Cohesin-mediated loop extrusion plays a fundamental role in the CSR mechanism (1, 14). In resting B cells, the intronic enhancer (iEµ), the closely linked Sµ and the downstream 30 kb long 3′RR function as dynamic, transcription-based loop anchors for cohesin-mediated loop extrusion between them (14). The loop extrusion process juxtaposes Sµ, iEµ and the 3′RR to form a nascent class-switch recombination-center (CSR-C) in naive B cells (14). Activation-induced transcription from a targeted I-promoter upstream of each acceptor S region (10), which is largely dependent on the 3′RR (12, 13), renders that targeted region a new dynamic loop extrusion impediment that leads to the targeted S region alignment with donor Sµ in the CSR-C (14). B cell activation also induces the AID expression, which targets DSBs in aligned, transcribed S region donor and acceptor S regions, followed by their deletional end-joining via a cohesin-mediated extrusion-based mechanism within the CSR-C (1, 11, 14).

Mouse V(D)J exon SHM also is targeted by transcription (3, 15). *Igh*, *Igκ* and *Igλ* variable region exons contain three complementarity-determining region (CDR) sequences that encode antigen-contact sites. CDRs contain AID targeting RGYW motifs, including AGCT motifs, at a much lower density than those in S regions (8). The *Igh* 3′RR enhancers and *Igκ* enhancers were also implicated in SHM, at least in part by promoting transcription through V(D)J exons to facilitate AID targeting (4, 16, 17). Homozygous deletion of the 3′RR abrogated low-level SHM of J_H_-iEµ intron sequences just downstream of the V(D)J exon, but only mildly reduced J_H_-iEµ intron transcription, suggesting that the 3′RR may have a role in promoting SHM beyond transcription per se (18–20). However, as these important studies did not examine SHM in V(D)J exons and also revealed that the 3′RR deletion reduced BCR expression, which might indirectly affect SHM (18), the precise role of the 3′RR in SHM remains to be fully determined. In mice, measurement of AID activity on a productively rearranged *Igh* V(D)J allele, encoding the VB1-8 heavy chain, versus a "nonproductive" VB1-8 passenger allele, identical to the productive allele except for an inactivating mutation, demonstrated that the V(D)J exon location is "privileged" for SHM targeting in GC B cells (8). In this regard, these studies demonstrated that diverse sequences, including bacterial gene sequences, undergo SHM in place of the passenger VB1-8 sequence (8). These findings emphasized the long-standing question of how AID is targeted to Sµ sequences, but not to closely linked V(D)J exon sequences during CSR (21). In this context, we proposed that in GC B cells, cohesin-mediated loop extrusion directly juxtaposes the 3′RR with the *Igh* variable region exon to establish a SHM center (SHM-C), that contributes to the privileged variable region exon location for SHM (14). We also noted that an active loop extrusion mechanism, as used for CSR, would likely be requisite for juxtaposing the 3′RR and V(D)J exon over the several hundred kilobases of chromosomal distance that separates them. Finally, this SHM-C model also proposed that I-region promoters would likely be down-regulated in GC B cells to allow direct juxtaposition of the 3′RR with the V(D)J exon in the absence of intervening transcriptional impediments activated for CSR (14). Loop extrusion also has been implicated in bringing enhancers and AID SHM targets together in other chromosome loop domains (22).

**Fig. 1.**
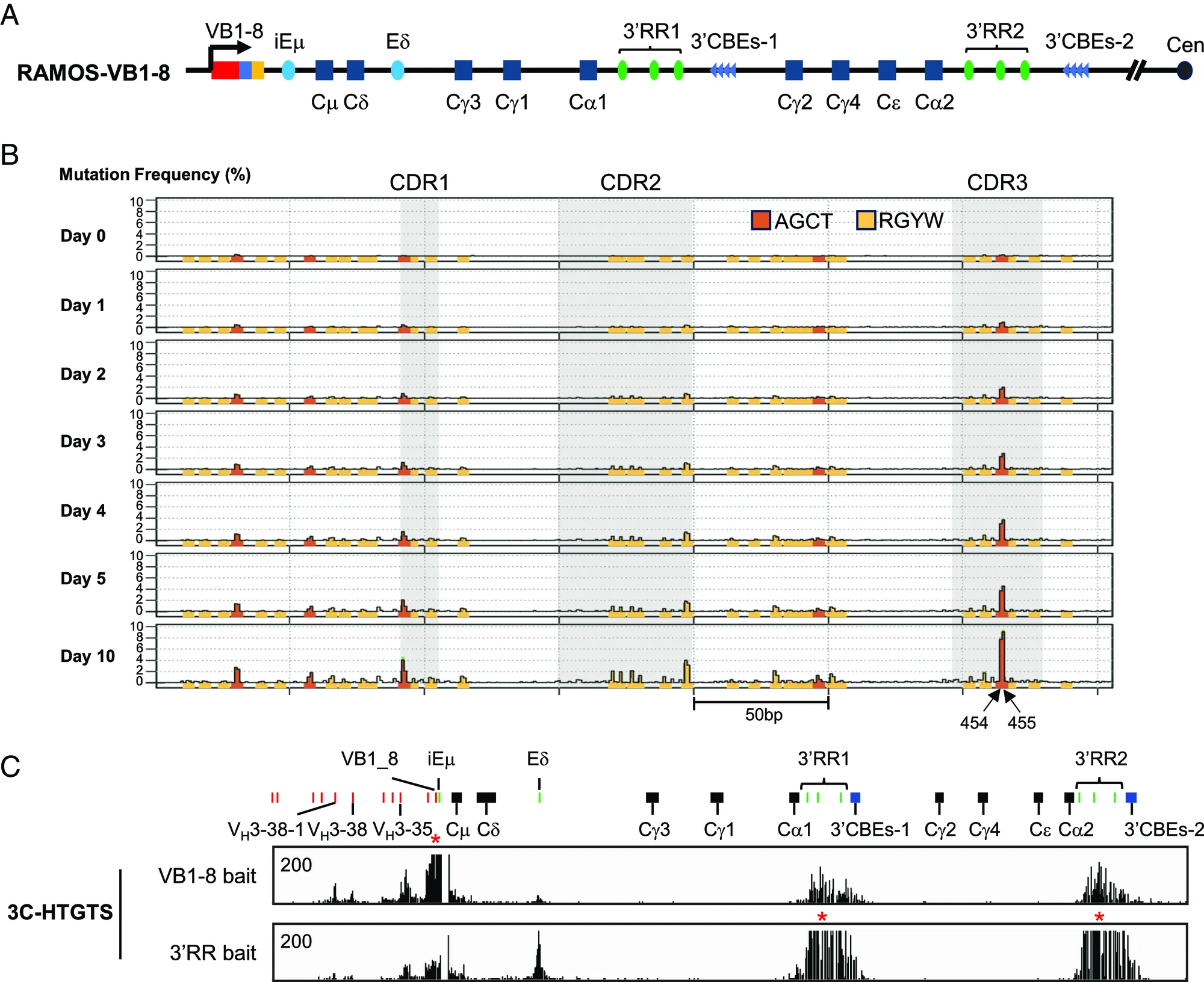
Chromatin organization and mutational landscape of the *IGH* locus in RAMOS-VB1-8 cells. (*A*) Schematic of the *IGH* constant region in the RAMOS-VB1-8 cell line. The endogenous rearranged human V(D)J was replaced with the mouse VB1-8 sequence. Individual enhancers within the 3′ regulatory regions (3′RRs) are depicted as green ovals. (*B*) Somatic hypermutation (SHM) profiles across the VB1-8 exon following doxycycline-induced AID expression at the indicated time points, as determined by SHM-Rep-Seq. The y-axis shows mutation frequency at each nucleotide position plotted as mean ± SEM from 3 independent repeats. Red and orange bars indicate AGCT and other RGYW motifs, respectively. (*C*) 3C-HTGTS interaction profiles across the 3′ *IGH* locus in RAMOS-VB1-8 cells, using VB1-8 and the 3′RRs as baits, respectively. Bait positions are indicated by red stars. All peaks are within the displayed scale range except for peaks at the bait sites. VB1-8 and upstream V_H_ segments are indicated by red lines; iEµ, Eδ, and individual 3′RR enhancers are indicated by green lines. Constant region exons are depicted as black boxes and 3′ CTCF-binding elements (3′CBEs) as blue boxes.

The human *IGH* C_H_ locus spans 300 kb downstream of the V(D)J exon and is organized differently from that of the mouse. Specifically, the human C_H_ locus is organized into two tandemly duplicated clusters: 5′–V(D)J–Cµ–Cδ–Cγ3–Cγ1–Cα1–3′RR1–3′CBEs-1–Cγ2–Cγ4–Cε–Cα2–3′RR2–3′CBEs-2–3′ (10, 23) (Fig. 1*A*). Both clusters have a 3′RR and adjacent 3′CBEs, which could, in theory, separate them into distinct domains (10, 23). Similar to the mouse, multiple B cell–specific *cis*-regulatory elements are present in the distal human C_H_ locus, including iEµ, between J_H_s and Sµ, an element downstream of Cδ referred to as Eδ (23), and the V(D)J exon promoter known to be critical for SHM in mice (15). Cell line models played a fundamental role in efforts to discover and characterize the role of chromatin loop extrusion in V(D)J recombination and *Igh* CSR (1). In particular, mutations can be introduced and impacts characterized rapidly. A large body of work over the past decades demonstrated the human Burkitt's lymphoma-derived RAMOS GC B cell line is a useful model for SHM studies (22–27). In RAMOS cells, the *IGH* C_H_ locus exists in two distinct allelic configurations. One allele retains a productive V4-34/D3-10/J6 exon, which undergoes constitutive SHM in its endogenous chromosomal location; while the second allele is involved in a chromosomal translocation between Sµ and c-*MYC*, leading to dysregulated c-*MYC* expression and SHM (28). In this regard, we previously discovered that the 3′RR can activate c-*Myc* translocations several hundred kb upstream into Sµ in mouse B cell lymphomas (29). In theory, the activation of SHM in both the RAMOS V(D)J exon and the translocated c-*MYC* gene (28) might occur through loop extrusion-based juxtaposition with the 3′RRs.

RAMOS cells were found to undergo increased SHM of their *IGH* and *IGL* variable region exons by introduction of a hyperactive AID mutant, AID7.3 (30). More recently, a further engineered RAMOS-based system was developed by deleting the endogenous AID gene (*AICDA*) and introducing a doxycycline-inducible AID7.3 cassette into the *AAVS1* locus, generating the AID7.3in cell line used for studies of the roles of enhancer-based transcription in stimulating SHM (26). The AID7.3in line was further engineered to generate the RASH RAMOS line which contained a SHM reporter that was used to identify HMCES as a DNA repair factor involved in SHM (31). The RASH cell line, as well as the CH12/F3 mouse B cell lymphoma line, was used to identify ELOF1 as a transcription elongation factor involved in SHM (32, 33). Another RAMOS system was developed in which endogenous *AICDA* was inactivated and the endogenous V(D)J exon replaced with other unmutated human V(D)J exons or the mouse VB1-8 V(D)J exon (27). In this system, SHM was induced by ectopic expression of a C-terminally truncated AID mutant and the introduced VB1-8 exon was found to have a similar SHM pattern to that in mouse GC B cells (27). However, in this study, roles of *cis*-regulatory element(s) in governing SHM were not addressed, leaving the relationship between SHM activity and transcriptional regulation an interesting unresolved question (27). Additional studies in this RAMOS line implicated existence of an "*IGH* hub" structure involving interactions of the V(D)J exon with the 3′RRs. This hub structure was detected 8 h after depletion of the RAD21 core cohesin factor in cycling RAMOS cells (23); However, studies of potential roles for the 3′RRs or the hub structure in SHM were not addressed in this study.

## Results

### A Modified Human RAMOS Cell Line System to Study SHM.

To test mechanisms and elements that control SHM, we further modified the previously described human AID7.3in RAMOS-derived cell line, in which the endogenous *AICDA* gene was deleted and replaced with a doxycycline (Dox)-inducible hyperactive AID mutant (AID7.3) targeted to the *AAVS1* locus (26). As the RAMOS *IGH* V4-34/D3-10/J6 expressed from its endogenous V4-34 promoter had already accumulated SHMs (24), we replaced it with the well-studied mouse VB1-8 exon that lacks any endogenous SHMs, allowing us to readily detect newly introduced SHMs (27). We previously characterized SHM patterns of the VB1-8 V(D)J exon in mouse GC B cells, as it served as the foundation for our mouse passenger-allele SHM studies (8). The unmutated, experimentally tractable V(D)J exon is transcribed from its own promoter and is also readily distinguishable from upstream human V_H_ segments in the various high-throughput assays used for our studies. For downstream studies described, we refer to the VB1-8 targeted AID7.3in cells with the endogenous *AICDA* gene deleted as the RAMOS-VB1-8 cells (Fig. 1*A*). Following Dox-induced AID expression, we analyzed mutation profiles of the VB1-8 allele using our unbiased, high-throughput SHM-Rep-Seq assay (34). Cycling RAMOS-VB1-8 cells accumulated substantial levels of SHM over a 10-d time course. During this time course, SHM frequencies steadily increased at AID hotspots including RGYW and AGCT motifs in both CDRs and framework regions (Fig. 1*B*). The overall SHM pattern was quite similar to that which we found for the mouse VB1-8 exon in mouse GC B cells (8). Overall, these results established that RAMOS-VB1-8 cell lines accumulate SHMs at AID hotspots over the tested time course, and that levels and patterns resembled those of the same VB1-8 exons in normal mouse GC B cells that had accumulated 3 to 10 SHMs (8).

### Establishment of a SHM Center in RAMOS Cells.

To investigate the chromatin architecture associated with SHM targeting, we performed high-resolution 3C-HTGTS analyses on RAMOS-VB1-8 cell lines, baiting from the VB1-8 region and the HS1,2 region in the 3′RRs. Given the high resolution of 3C-HTGTS (35), we identified clear and robust interactions between the VB1-8 exon and both 3′RR1 and 3′RR2 (Fig. 1 *C*, *Top* panel). These interactions were broadly distributed across both 3′RRs including regions containing HS3, HS1,2 and HS4 (Fig. 1 *C*, *Top* panel). In analogy to interactions between 3′RR and V(D)J exon observed during CSR (14), the broad interaction patterns between VB1-8 and the 3′RRs are consistent with the 3′RRs serving as dynamic impediments to loop extrusion. The VB1-8 region also interacted, to a much lesser extent, with both 3′CBEs and with a putative enhancer element termed Eδ (23), located approximately 20 kb downstream of Cδ, that is not conserved in the mouse C_H_ locus (10) (Fig. 1 *C*, *Top* panel). Reciprocally, baiting from the HS1,2 region in both 3′RRs, which have greater than 90% sequence homology that obviates ability to distinguish them by sequence (36), we confirmed that 3′RRs broadly interact with VB1-8 locale and those of iEµ and Eδ (Fig. 1 *C*, *Bottom* panel). In contrast to mouse B cells activated for CSR (14), very few interactions were found with any other sequence regions including those containing S regions and C_H_ exons with VB1-8 and 3′RRs probes (Fig. 1 *C*, *Top* and *Bottom* panels). Together, these findings establish a human SHM model system that recapitulates many key features of the proposed in vivo SHM-C and reveal a network of long-range chromatin interactions linking the VB1-8 exon, iEµ, Eδ and the 3′RRs. These interactions are similar to those of the previously proposed *IGH* Hub elucidated at lower resolution (23). Overall, these observations are consistent with these implicated regulatory elements cooperatively forming a "SHM-C" that promotes efficient AID targeting and SHM, and that is structurally unique from that of a CSR-C that forms over the same region in B cells activated for CSR (14). The absence of other S region loop extrusion impediments is also consistent with the proposal that S region associated impediments are down-regulated in GC B cells to support unimpeded loop extrusion between the proximal 3′RR and the V(D)J exon to form a SHM-C (14).

### Combined Regulation of SHM by 3′RRs.

**Fig. 2.**
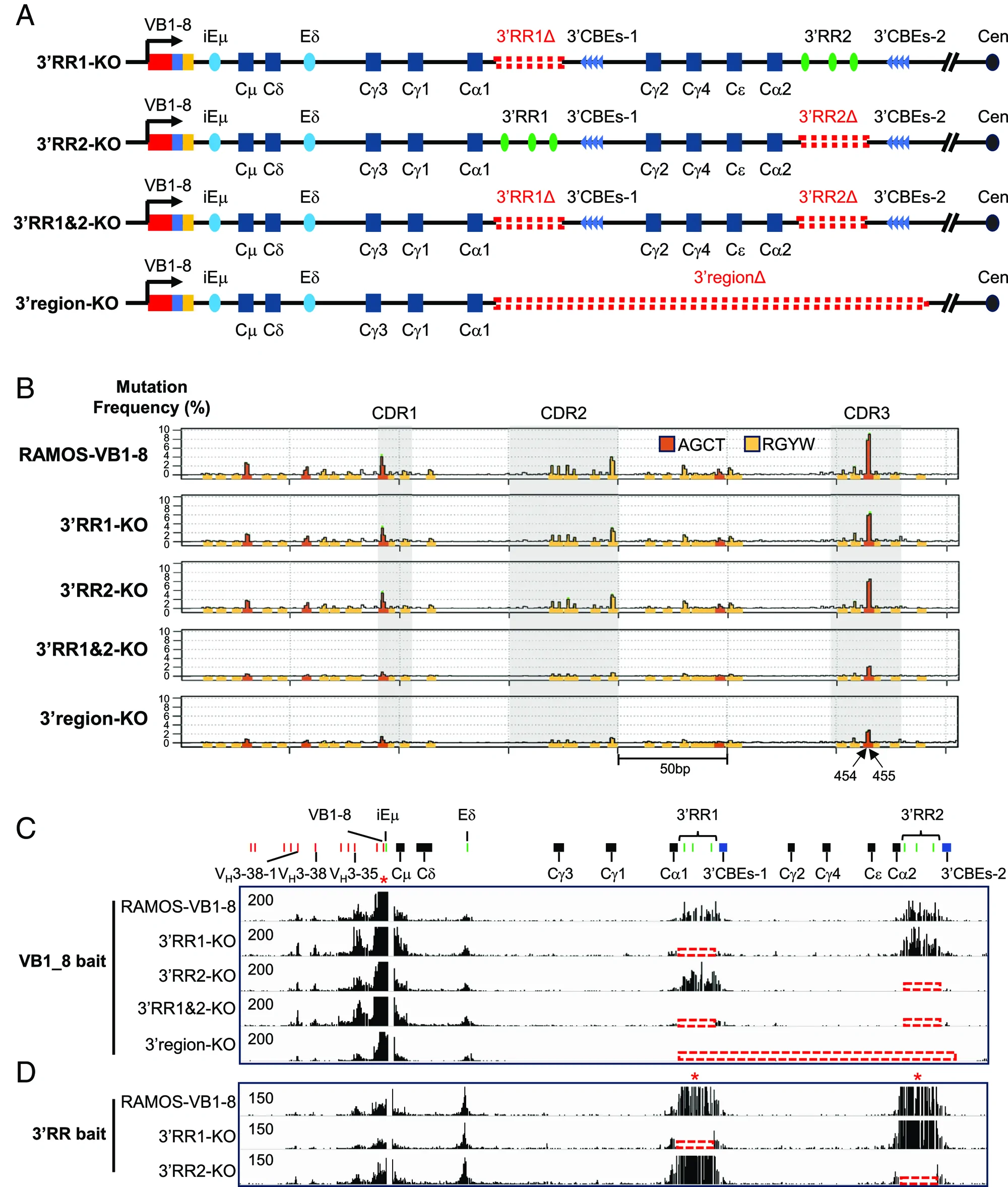
Impact of 3′RR deletions on SHM and chromatin interactions at the *IGH* locus. (*A*) Schematics of the *IGH* constant region in 3′RR1-KO, 3′RR2-KO, 3′RR1&2-KO, and 3′region-KO cell lines, illustrating the extent of each deletion. (*B*) SHM profiles across the VB1-8 exon in RAMOS-VB1-8, 3′RR1-KO, 3′RR2-KO, 3′RR1&2-KO, and 3′region-KO cells following 10 d of doxycycline-induced AID expression, as determined by SHM-Rep-Seq. Data are plotted as mean ± SEM from 3 independent repeats. RAMOS-VB1-8 and 3′RR1&2-KO SHM patterns are highly similar (two-sided Pearson's *r* = 0.98, *P* = 3.9 × 10^−252^). RAMOS-VB1-8 and 3′region-KO SHM patterns are highly similar (two-sided Pearson's *r* = 0.96, *P* = 3.04 × 10^−195^). The SHM profiles from Fig. 1*B* (Day 10) are reproduced here as control to facilitate comparison. (*C*) 3C-HTGTS interaction profiles across the 3′ *IGH* locus in RAMOS-VB1-8, 3′RR1-KO, 3′RR2-KO, 3′RR1&2-KO, and 3′region-KO cells, using VB1-8 as bait. (*D*) 3C-HTGTS interaction profiles across the 3′ *IGH* locus in RAMOS-VB1-8, 3′RR1-KO, and 3′RR2-KO cells, using the 3′RRs as bait.

To investigate the relative contribution of the two human 3′RRs to SHM, we generated a series of knockout cell lines, including lines with a 3′RR1-KO, a 3′RR2-KO, a 3′RR1/2-double KO (3′RR1&2-KO), and a larger 3′-region deletion encompassing both regulatory regions (“3′region-KO”) (Fig. 2*A*). Deletion of either individual 3′RR in the RAMOS-VB1-8 line caused at most a modest reduction in SHM frequency (Fig. 2*B* and *SI Appendix*, Fig. S1). Correspondingly, 3C-HTGTS-based chromatin interaction analyses revealed that the remaining intact 3′RR maintained robust long-range interactions with the VB1-8 region (Fig. 2 *C* and *D*). Overall, these results demonstrated that each individual 3′RR is independently able to support efficient SHM in the absence of the other. In contrast, simultaneous deletion of both 3′RRs resulted in a substantial reduction in SHM frequency without affecting the pattern, but with still significant residual levels, confirming that the duplicated human 3′RRs function redundantly to promote efficient SHM (Fig. 2*B* and *SI Appendix*, Fig. S1). Notably, deletion of the entire downstream region containing the two 3′RRs and both 3′CBEs did not further reduce residual SHM activity (Fig. 2*B* and *SI Appendix*, Fig. S1). 3C-HTGTS analyses, baiting from the VB1-8 locus, reveal that VB1-8 interacts similarly with each 3′RR in the absence of the other and continues to interact with Eδ in the absence of both 3′RRs, suggesting a potential role of either iEµ, Eδ, or both in maintaining residual SHM activity (Fig. 2*C*).

### 3′RRs Promote AID Targeting Independent of Activating Transcription.

**Fig. 3.**
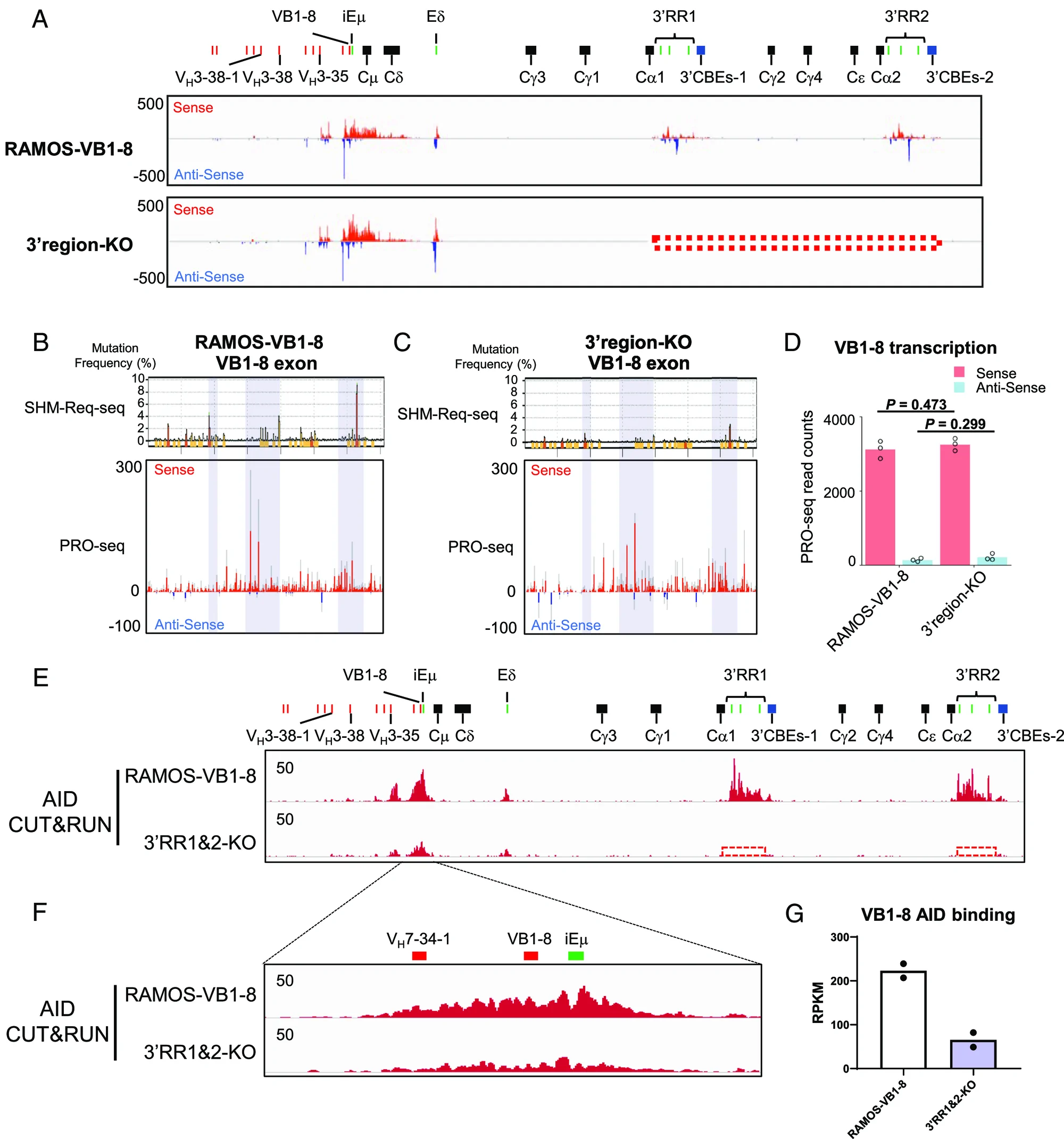
Transcriptional activity and AID occupancy at the *IGH* locus. (*A*) PRO-seq profiles across the *IGH* constant region in RAMOS-VB1-8 and 3′region-KO cells. (*B* and *C*) SHM profiles and normalized PRO-seq read counts across the VB1-8 exon in RAMOS-VB1-8 (*B*) and 3′ region-KO (*C*) cells. The SHM profiles from Fig. 2*B* (RAMOS-VB1-8 and 3′region-KO) are reproduced here to facilitate comparison. PRO-seq data represent the mean ± SD of three independent repeats. SHM profiles are plotted as mean ± SEM from 3 independent repeats. (*D*) Normalized PRO-seq read counts across the VB1-8 in RAMOS-VB1-8 and 3′region-KO cells. Data represent the mean of three independent repeats. Statistical significance was assessed using a two-tailed unpaired Student's *t* test. (*E* and *F*) AID CUT&RUN profiles across the *IGH* constant region (*E*) and VB1-8 locus (*F*) in RAMOS-VB1-8 and 3′RR1&2-KO cells. (*G*) RPKM values of AID CUT&RUN signal across the VB1-8 in RAMOS-VB1-8 and 3′RR1&2-KO cells. Data represent the mean of two independent repeats.

To test for potential mechanisms by which the 3′RRs promote SHM of V(D)J exons, we assessed their role in inducing or maintaining VB1-8 exon transcription, by performing PRO-seq in RAMOS-VB1-8 and the 3′region-KO cells in the absence of AID induction (Fig. 3*A*). In RAMOS-VB1-8 cells, transcription was initiated from the VB1-8 promoter and extended through the downstream intronic region containing iEµ, Sµ, Cµ and the Cδ exons (Fig. 3 *A*, *Top* panel). Further downstream, Eδ and both 3′RRs also exhibited robust transcriptional activity (23). Notably, these transcriptionally active regions corresponded closely to the chromatin interaction network identified by our 3C-HTGTS analyses, suggesting coordinated organization of transcriptional and structural regulatory elements within the SHM-C (Fig. 1*C*). Surprisingly, the 3′region-KO cells retained robust transcription over the VB1-8, iEµ, Sµ and the Cµ and Cδ exons at levels comparable to those in RAMOS-VB1-8 cells (Fig. 3 *A*, *Bottom* panel). Within the VB1-8 locus, the rearranged V(D)J exon in both RAMOS-VB1-8 and 3′region-KO cells exhibited strong sense transcription accompanied by relatively low levels of antisense transcription (Fig. 3 *B* and *C* and *SI Appendix*, Fig. S2 *A* and *B*). Thus, deletion of the entire 3′ region had little effect on the quantitative transcriptional output across the VB1-8 region (Fig. 3*D*), despite causing a profound reduction in SHM frequency (Fig. 3 *B* and *C*, *Top* panels). These findings indicate that loss of the 3′RRs does not appreciably alter transcription levels prior to AID targeting and support early findings that suggest the mouse 3′RRs can promote SHM through a transcription-level independent mechanism (18).

To investigate whether reduced SHM resulted from impaired AID recruitment in 3′RR1&2-KO cells, we performed AID CUT&RUN. AID accumulated robustly across the VB1-8 exon (Fig. 3 *E* and *F*, *Top* panels) as well as other transcribed sequences at Eδ and 3′RRs in RAMOS *IGH* 3′ region (Fig. 3 *E* and *F*, *Top* panels). In contrast, deletion of both 3′RRs markedly reduced AID occupancy across the VB1-8 exon region (Fig. 3 *E* and 3 *F*, *Bottom* panels, and Fig. 3*G*). Together, these results indicate that the 3′RRs facilitate efficient AID recruitment and/or retention at the V(D)J exon, beyond any mechanisms involving transcription-level-mediated effects proposed to increase AID substrate targeting ability (37).

### Robust AID-Induced SHM over a 10-d Time Course in G1-Arrested RAMOS Cells.

**Fig. 4.**
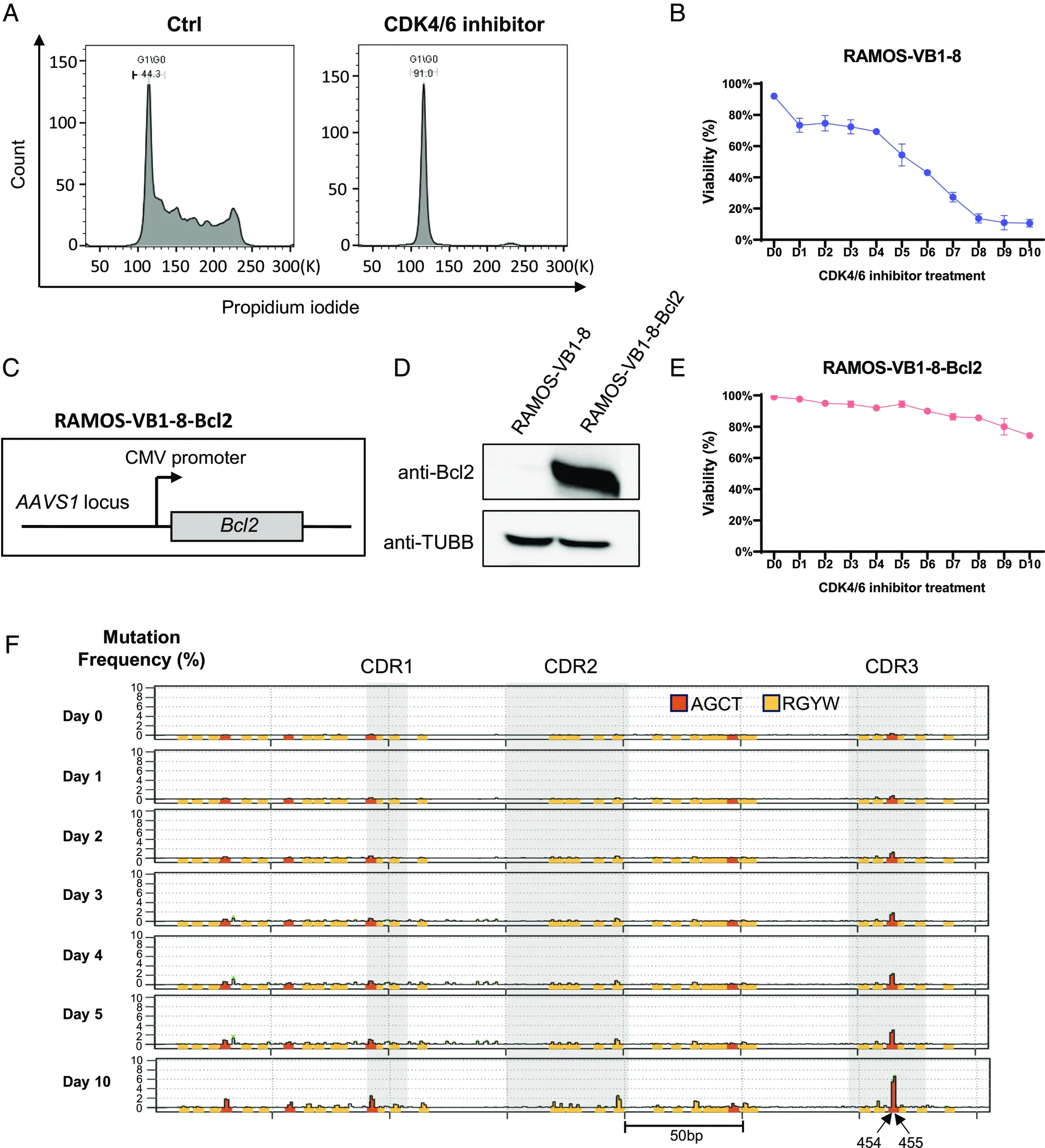
Establishment and validation of a cell cycle-arrested system for SHM analysis. (*A*) Flow cytometry of propidium iodide-stained RAMOS-VB1-8 cells with or without CDK4/6 inhibitor treatment. (*B*) Cell viability of RAMOS-VB1-8 cells following 10 d of CDK4/6 inhibitor treatment. Data represent the mean ± SD of three independent repeats. (*C*) Schematic of the *AAVS1* locus depicting the integrated CMV-*Bcl2* overexpression cassette in RAMOS-VB1-8-Bcl2 cells. (*D*) Western blot analysis of Bcl2 protein levels in RAMOS-VB1-8 and RAMOS-VB1-8-Bcl2 cells. Raw data are shown in *SI Appendix*, Fig. S8*A*. (*E*) Cell viability of RAMOS-VB1-8-Bcl2 cells following 10 d of CDK4/6 inhibitor treatment. Data represent the mean ± SD of three independent repeats. (*F*) SHM profiles across the VB1-8 exon in G1-arrested RAMOS-VB1-8-Bcl2 cells following doxycycline-induced AID expression at the indicated time points, as determined by SHM-Rep-Seq. Data are plotted as mean ± SEM from three independent repeats.

A series of mouse studies have implicated SHM as predominantly occurring in the G1 cell cycle phase (38–43). We sought to test the ability of AID to induce SHMs in G1-arrested RAMOS cells. We found that the PD0332991 (“CDK4/6 inhibitor”) efficiently arrested RAMOS cells in the G1 phase, thereby providing a system to examine SHM under G1-arrest conditions (Fig. 4*A*). However, prolonged G1 arrest resulted in a substantial loss of cell viability over the course of CDK4/6 inhibitor treatment (Fig. 4*B*). To improve viability during extended G1 arrest, we introduced a *Bcl2* overexpression cassette into the *AAVS1* locus of the RAMOS-VB1-8 cells (Fig. 4 *C* and *D*). Hereafter, we refer to the Bcl2-overexpressing RAMOS-VB1-8 cells as RAMOS-VB1-8-Bcl2 cells. RAMOS-VB1-8-Bcl2 cells maintained high viability even after 10 d of CDK4/6 inhibitor treatment (Fig. 4*E*). Strikingly, G1-arrested RAMOS-VB1-8-Bcl2 cells remained capable of undergoing robust SHM following AID induction. Moreover, the G1-arrested RAMOS-VB1-8-Bcl2 cells accumulated SHMs over the 10-d test period with a similar pattern to that of cycling RAMOS-VB1-8 cells (Figs. 1*B* and 4*F*). The final SHM frequencies were very modestly reduced compared with those in cycling cells (Figs. 1*B* and 4*F* and *SI Appendix*, Fig. S3).

### SHM Occurs in G1-Arrested RAMOS Cells After RAD21 Depletion.

To test potential roles of cohesin-mediated loop extrusion in assembly of the SHM-C (14), we sought to deplete the RAD21 cohesin ring component in RAMOS cells. However, RAD21 inactivation generates a very rapid decrease in cycling mammalian cell viability (44, 45). In that regard, we previously reported that we can viably deplete numerous different cohesin components, including RAD21, in G1-arrested, v-*Abl*-transformed pro-B cells that ectopically express Bcl2 (46, 47). Based on these findings, we generated a homozygous RAD21 degron cell line in RAMOS-VB1-8-Bcl2 cells by tagging both endogenous RAD21 alleles with a FKBP12^F36V^ degron cassette (*SI Appendix*, Fig. S4*A*) (48). Hereafter, we refer to the RAMOS-VB1-8-Bcl2 cells with homozygous RAD21 degron as RAD21-degron RAMOS cells. Western blot analysis confirmed efficient depletion of RAD21 protein in RAD21-degron RAMOS cells within 6 h of dTAGV-1 treatment (*SI Appendix*, Fig. S4*B*). Under G1 arrest, RAD21-depleted cells maintained nearly 80% viability for 4 d and approximately 60% viability at 10 d (*SI Appendix*, Fig. S4*C*), providing a system for investigating long-term effects of RAD21 depletion on SHM and the SHM-C architecture.

**Fig. 5.**
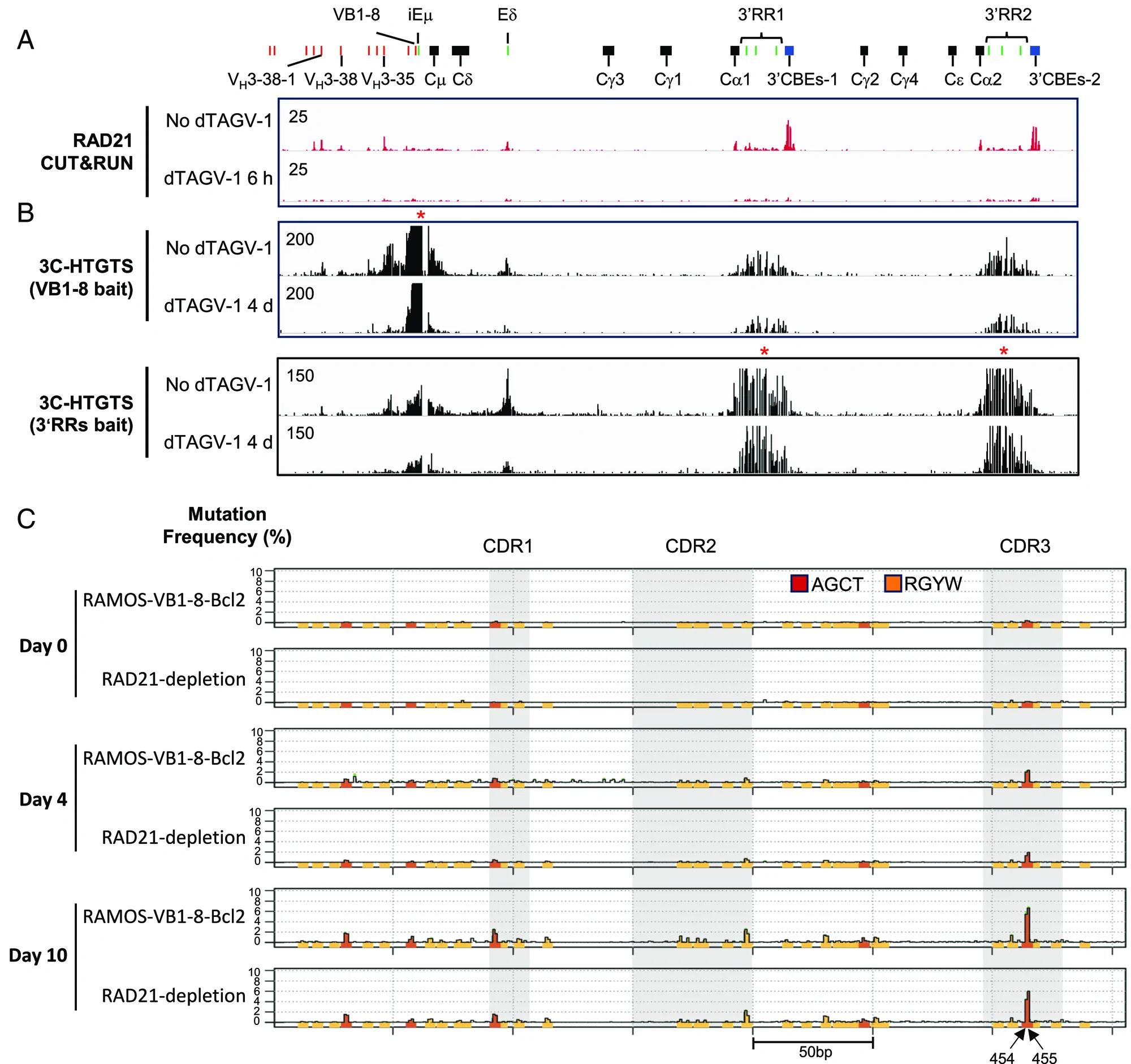
Effects of RAD21 depletion on chromatin organization and SHM at the *IGH* locus. (*A*) CUT&RUN profiles of RAD21 occupancy across the *IGH* locus in RAD21-degron cells treated with or without dTAGV-1 for 6 h. (*B*) 3C-HTGTS interaction profiles across the 3′ *IGH* locus in RAD21-degron cells treated with or without dTAGV-1 for 4 d, using VB1-8 and 3′RRs as baits, respectively. (*C*) SHM profiles across the VB1-8 exon in G1-arrested RAMOS-VB1-8-Bcl2 and RAD21-depleted cells following doxycycline-induced AID expression at the indicated time points, as determined by SHM-Rep-Seq. Data are plotted as mean ± SEM from three independent repeats. RAMOS-VB1-8-Bcl2 and RAD21-depletion SHM patterns are highly similar (Day 4: two-sided Pearson's *r* = 0.86, *P* = 4.59 × 10^−106^; Day 10: two-sided Pearson's *r* = 0.99, *P* = 2.44 × 10^−292^). The SHM profiles from Fig. 4*F* (Days 0, 4, and 10) are reproduced here to facilitate comparison.

To define the genomic distribution of cohesin, we performed CUT&RUN for RAD21 (Fig. 5*A*). Before dTAGV-1 treatment, RAD21 was highly enriched at both 3′CBEs, consistent with their role as loop anchors. Lower levels of RAD21 accumulation were detected at the 3′RRs, Eδ, and several CBE sites, convergent to the 3′CBEs, within the upstream V_H_ locus region proximal to the VB1-8 exon. Accumulation of cohesin at these elements, which all interact with the VB1-8 exon and 3′RRs based on 3C-HTGTS assays (Figs. 1*C* and 5*A*), is consistent with cohesin involvement in establishment of the SHM-C complex via loop extrusion. As expected, chromatin-associated RAD21 accumulation was essentially absent following 6 h of dTAGV-1 treatment (Fig. 5 *A*, *Bottom* panel). To examine effects of RAD21 depletion on chromatin interactions genome-wide, RAD21-degron cells were treated with or without dTAGV-1 for 6 h before G1 arrest and AID induction, and after 4 d of RAD21 depletion, cells were harvested for Hi-C analysis. The *IGH* 3′ region contains two sets of homologous constant regions with high sequence similarity, and contains very highly repetitive sequences in the duplicated switch regions, 3′RRs, and 3′CBEs, which creates challenges for mapping Hi-C reads in this region (*SI Appendix*, Fig. S4*D*; see legend for details). Even so, the Hi-C results showed that most chromatin loops and topologically associating domains (TADs) were eliminated genome-wide (*SI Appendix*, Fig. S4*E*), confirming the effective depletion of RAD21.

To determine the contribution of cohesin to SHM-associated chromatin architecture, we examined chromatin interactions within the *IGH* locus at high resolution 4 d after RAD21 depletion via 3C-HTGTS that employed the VB1-8 region and 3′RRs as baits. Remarkably, despite the absence of most chromatin loop domains genome-wide, long-range interactions between VB1-8 and the downstream 3′RRs were reduced but still substantially preserved after 4 d of RAD21 depletion (Fig. 5*B* and *SI Appendix*, Fig. S4*F*). This finding indicates that the VB1-8–3′RR-based SHM-C is maintained relatively intact for at least 4 d in the absence of cohesin-mediated loop extrusion in G1-arrested RAMOS cells (Fig. 5*B* and *SI Appendix*, Fig. S4*F*). In contrast, interactions between VB1-8 and CTCF sites in the proximal upstream V_H_ locus region were markedly decreased (Fig. 5*B* and *SI Appendix*, Fig. S4*F*). To determine whether cohesin loss affected transcription, we performed PRO-seq following 4 d of RAD21 depletion. Transcription levels across the VB1-8 region and the downstream Eδ and 3′RRs were basically unchanged in the absence of RAD21 (*SI Appendix*, Fig. S5 *A*–*D*). Consistent with the persistence of both VB1-8–3′RR-based SHM-C structure and its transcriptional activity, SHM-Rep-Seq analysis showed that RAD21 depletion in the G1-arrested RAD21-degron cells had essentially no effect on SHM accumulation in the VB1-8 exon, as compared to that of the G1-arrested RAMOS-VB1-8-Bcl2 cells or RAD21-degron cells without dTAGV-1 treatment. (Fig. 5*C* and *SI Appendix*, Fig. S6 *A* and *B*). Together, these results demonstrate that ongoing cohesin-mediated loop extrusion is largely dispensable for maintenance of the architectural organization, transcriptional activity, and functional SHM activity of the SHM-C over a multiday period in G1-arrested RAMOS cells. In contrast to our findings, a recent cycling RAMOS cell study, published shortly after this study was submitted, reported that both 3′RR interactions with the endogenous human V(D)J exon and SHM were abrogated within 24 h after RAD21 depletion (49). The difference between our findings and those of this other study may reflect studies being done in cycling versus G1-arrested cells. For example, the putative SHM-C may be rapidly disassembled when G1-arrested cells enter the cell cycle and initiate DNA replication, an interesting possibility that could be readily tested in our G1-arrestable model.

## Discussion

The human RAMOS GC B cell lymphoma line has provided an important in vitro model system for studying the mechanism of SHM (22–27). For the current studies, we modified the AID7.3in RAMOS cell line in which the endogenous AID gene had been deleted and an inducible hyperactive AID7.3 mutant was introduced (26). We further replaced the endogenous human *IGH* V(D)J exon with an unmutated mouse VB1-8 V_H_(D)J_H_ exon (Fig. 1*A*), for which we previously characterized the SHM pattern in depth in mouse GC B cells (8). We used SHM-Rep-Seq to characterize SHM levels and patterns upon AID induction over a 10-d time course in the RAMOS-VB1-8 line (Fig. 1*B*). We found that the RAMOS VB1-8 exon accumulated SHMs mainly in AID-targeting motifs with a pattern similar to that of GC B cells that have accumulated relatively low levels of SHM (Fig. 1*B*) (8). Indeed, mouse GC B cell SHM levels reach much higher levels than those observed over the 10-d time course in the RAMOS-VB1-8 cell line (8). One possible explanation for lower-level SHM in RAMOS-VB1-8 cells is that AID activity may be rate-limiting for potential reasons discussed below. Another is that high-level SHM accumulation in mouse GC B cells can involve multiple rounds of B cell reentry into the GC (50). In theory, we could address this latter possibility through several rounds of AID induction in our G1-arrestable RAMOS-VB1-8 model. In addition, recent studies reported that incorporation of a LINE-1 element upstream of the RAMOS endogenous V(D)J exon promoter enhances SHM, which could provide a potential strategy to further increase SHM frequencies in our RAMOS-VB1-8 system (51).

We employed 3C-HTGTS to test our hypothesis that a SHM-C, analogous to the CSR-C (14), provides a privileged location for the accumulation of SHMs (Fig. 1*C*) (14). Similar to the CSR-C in mouse CSR-activated B cells (14), the V(D)J exon in RAMOS cells robustly interacts across the 20-kb length of both 3′RRs, and also with other putative elements, such as the Eδ enhancer (Fig. 1*C*) (14, 23). Notably, deletion of one or the other of the 3′RRs had no obvious effect on the interactions of VB1-8 with the other remaining 3′RR and had, at most, modest effects on SHM over the 10-d time course (Fig. 2 *B* and *C*). On the other hand, deletion of both 3′RRs or the entire region containing them and their associated 3′CBEs reduced SHM levels by approximately 4-fold but did not alter SHM patterns (Fig. 2*B* and S1). These findings indicate that either human 3′RR can form a functional SHM-C in the absence of the other, but in the absence of both, SHM is greatly reduced, but not eliminated. Notably, we found that deletion of both 3′RRs reduced AID occupancy across the VB1-8 exon, supporting a role for the 3′RRs in promoting AID recruitment (Fig. 3 *E*–*G*) (16). These observations are consistent with the SHM-C model, in which chromatin loop extrusion brings 3′RRs into proximity with the V(D)J exon, thereby facilitating recruitment or retention of AID and/or AID-associated transcriptional regulators (14, 16, 22, 32, 33, 52).

PRO-seq analyses showed that complete deletion of the 3′RRs and associated sequences did not markedly alter transcription levels across the VB1-8 exon, despite the major effect that this deletion had on SHM (Fig. 3 *A*–*D*). We note that maintenance of overall transcription levels in the dual 3′RR-deleted RAMOS lines does not exclude changes in other transcriptional features, such as RNA polymerase II (Pol II) pausing or stalling within the VB1-8 exon. In this regard, previous studies suggest that Pol II pausing/stalling facilitates AID targeting (16, 32, 33, 53, 54). Thus, additional studies will be required to determine whether and if so, how the dual 3′RR deletion allows ongoing transcription but alters transcriptional dynamics (e.g., Pol II pausing/stalling) and/or the recruitment of transcription-associated factors, which could impair both AID accumulation and AID transcription-targeted activity across the *IGH* V(D)J exon. Recent studies demonstrated that S region transcripts recruit AID to the *Igh* CSR-C to initiate CSR (7). As V(D)J exons lack dense arrays of transcribed AID targeting motifs present in S regions, any role for V(D)J exon transcripts in recruiting AID into a SHM-C would likely employ a different, yet unknown, mechanism.

The 3′CBEs have been proposed to serve as a major loop anchor within the mouse *Igh* locus (14, 55). We note that the RAMOS VB1-8 exon retained robust interactions with the 3′RRs even though the 3′CBEs-1 cluster is positioned between the two 3′RRs (Fig. 1*C*). This finding suggests that the intervening 3′CBEs-1 cluster does not block communication between the VB1-8 exon and the 3′RRs. One possibility is that these CBEs anchor convergent chromatin loops, thereby bringing the VB1-8 exon into close spatial proximity with both 3′RRs (See Model in *SI Appendix*, Fig. S7). Another possibility is that the impediment activity of the human 3′CBEs, which only have 4 CBE copies, is less than that of the mouse, which have 10 CBE copies (1). It is also theoretically possible that the 3′CBE impediment activity is functionally attenuated or neutralized in RAMOS cells, as previous studies have shown that CBE function can be modulated during B cell development (47).

A major innovation of our RAMOS-VB1-8 model was our finding that enforced Bcl2 overexpression allowed RAMOS-VB1-8-Bcl2 cells to be viably G1-arrested in the G0/G1 stage for up to 10 d after AID induction (Fig. 4 *A*–*E*), during which they accumulated VB1-8 mutations to a similar level and pattern as cycling RAMOS cells over the 10-d time course (Fig. 4*F*). This finding is of interest given prior cell cycle studies of AID activity in B cells. In this regard, it has been suggested that nuclear AID is more stable during the G1 stage (39). In addition, AID has been shown to undergo active nuclear import (56). Recent in vivo studies reported that GC B cells exhibit higher mutation efficiency during the G0/G1 stage (41, 42). Yet, whether AID is actually enriched in the nucleus during G1 arrest remains controversial (40, 56, 57). We note that our G1-arrested RAMOS-VB1-8-Bcl2 cells did not display higher SHM efficiency than cycling cells (*SI Appendix*, Fig. S3). Potential differences between our RAMOS findings and those of other studies in mouse GC B cells may reflect differences in cell types, experimental systems, or imaging strategies (40–42, 56, 57). However, our G1-arrestable RAMOS system should provide a useful system for pursuing single molecule/single cell imaging studies (7) to address relationships between nuclear accumulation of AID in the SHM-C and influences on SHM activity across the cell cycle.

Based on our previous studies showing that the CSR-C (14) and V(D)J recombination center (6, 35) can serve as broad and dynamic loop-extrusion anchors to bring long-range elements into proximity, we speculate that loop extrusion contributes to the initial establishment of the SHM-C. As discussed above, analogies of several aspects of our findings in this study to aspects of the generation of CSR-C support the notion that the SHM-C may be formed through cohesin-mediated loop extrusion between the 3′RRs and upstream transcriptional complexes in the V(D)J exon locale. In this regard, other extrusion impediments, such as the 3′CBEs and convergent CBEs upstream of the V(D)J exon locale may contribute to or enforce the assembly of a SHM-C (14, 55). However, unlike a nascent CSR-C, that requires secondary activation-based I-region promoter generated extrusion event to complete its structure, the postulated SHM-C, as we show here, would be preformed in GC B cells and only need induction of AID to generate its activity.

The G1-arrested RAMOS-VB1-8-Bcl2 cell SHM model, similar to our findings with G1-arrested *v-Abl* cell models (46, 47), can be viably depleted of RAD21 to test the potential roles of RAD21, and ongoing forward, other cohesin complex subunits or associated factors in the SHM process (*SI Appendix*, Fig. S4). Notably, four days of cohesin depletion did not abolish the VB1-8–3′RR interaction, indicating that, once established, maintenance of SHM-C interactions in G1-arrested cells is largely independent of ongoing cohesin-mediated loop extrusion (Fig. 5). Additional factors/mechanisms that could contribute to the maintenance of the SHM-C in viably G1-arrested RAMOS cells may include processed long noncoding RNAs (lncRNAs) (58), Mediator-associated interactions (59), and architectural transcription factors (60). Future studies using our G1-arrestable system will be critical for defining the mechanisms that stabilize the SHM-C after its establishment and the potential role of SHM-C stability in the GC reaction in vivo.

Finally, while our studies have implicated cohesin-mediated loop extrusion in the establishment of a SHM-C, the question of how such a SHM-C provides a "privileged" location for SHM targeting in GC B cells remains unanswered. The G1-arrestable RAMOS cell SHM model we describe should be useful for addressing this lingering question.

## Materials and Methods

### Cell Culture.

RAMOS B cells were cultured in RPMI-1640 medium, supplemented with 10% FBS and 1X Penicillin–Streptomycin–Glutamine. For induction of AID expression, cells were treated with 200 ng/mL doxycycline (Dox) for the indicated durations. For CDK4/6 inhibitor-induced G1 arrest, cells were treated with 10 μM PD0332991 for the indicated durations. For RAD21 depletion, RAD21-degron cells were first treated with 500 nM dTAGV-1 for 6 h, followed by treatment with 10 μM PD0332991 and 200 ng/mL Dox for the indicated durations prior to collection and analysis.

### Generation of Mutant RAMOS Cell Lines.

All cell lines were generated using a CRISPR/Cas9 strategy. For cell lines targeting the *IGH* constant region specifically, 3C-HTGTS using the VB1-8 and c-*MYC* regions as baits was performed to confirm that editing occurred exclusively at the VB1-8 allele rather than the translocated allele. sgRNA sequences used for cell line generation are listed in *SI Appendix*, Table S1.

### Flow Cytometric Analysis.

The distribution of cells across the cell cycle was assessed by propidium iodide (PI) staining, followed by flow cytometry. Cells were treated with or without 10 μM PD0332991 for 24 h, then collected and washed once with 1× DPBS. Cells were fixed in 70% ethanol at −20 °C for 24 h, pelleted by centrifugation, and resuspended in 1× DPBS containing 100 μg/mL RNase A. Following incubation at 37 °C for 30 min, cells were pelleted, resuspended in 1× DPBS containing 10 μg/mL PI, and incubated on ice for 10 min. Samples were then analyzed on a BD FACS Calibur flow cytometer (BD Biosciences). Data were analyzed using FlowJo software (v10). Intact cells were gated based on FSC-A/SSC-A parameters, and single cells were isolated using an FSC-A/FSC-H gate. Cell cycle distribution was determined based on PI fluorescence intensity.

### SHM-Rep-Seq and Data Analysis.

SHM-Rep-Seq libraries were prepared using the VB1-8 locus as bait, and the procedure was conducted as previously described (61). Libraries were sequenced on a NextSeq 2000 (Illumina) with 300-bp paired-end reads. SHM-Rep-Seq data were analyzed as previously described (8). Bio primers and red primers are listed in *SI Appendix*, Table S2.

### 3C-HTGTS and Data Analysis.

3C-HTGTS libraries were prepared as described (35). Libraries were sequenced on a NextSeq 2000 (Illumina) with 300-bp paired-end reads. Bio primers and red primers are listed in *SI Appendix*, Table S2. The 3C-HTGTS data were analyzed as previously described by aligning to hg38 reference genome containing the engineered *IGH* allele (35). For each figure, libraries were normalized to the same number of junctions prior to visualization as previously described (35).

### PRO-Seq and Data Analysis.

PRO-seq experiments were performed as previously described (62). Libraries were sequenced on a NextSeq 2000 (Illumina) with 50-bp paired-end reads. PRO-seq data were aligned to a modified hg38 reference genome containing the engineered *IGH* allele using Bowtie2 (v2.4.2). BAM files were sorted and indexed using SAMtools (v0.1.19). Strand-specific PRO-seq read counts were generated from the resulting BAM files using BEDTools (v2.29.0) and bamCoverage (v3.3.1) with a 1-bp bin size. For each figure, strand-specific PRO-seq read counts were normalized to the same total number of mappable reads as previously described (47). Normalized PRO-seq read counts from three independent repeats were averaged and visualized with error bars using a modified version of Integrative Genomics Viewer (IGV; https://github.com/Yyx2626/igv).

### CUT&RUN and Data Analysis.

CUT&RUN experiment was performed using a CUT&RUN Library Prep Kit (EpiCypher, 14-1048) according to the manufacturer's instructions. Libraries were sequenced on a NextSeq 2000 (Illumina) with 150-bp paired-end reads. The antibodies used for CUT&RUN are listed in *SI Appendix*, Table S3.

CUT&RUN data were aligned to a modified hg38 reference genome containing the engineered *IGH* allele using Bowtie2 (v2.4.2). BAM files were sorted and indexed using SAMtools (v0.1.19). Normalized coverage tracks were generated from the resulting BAM files using bamCoverage (v3.3.1) and CPM normalization. AID CUT&RUN experiments were performed in RAMOS-VB1-8 and independently generated 3′RR1&2-KO cells, in which both 3′RR1 and 3′RR2 were deleted while the intervening constant-region sequences were retained. The 3′region-KO and 3′RR1&2-KO lines showed comparable reductions in SHM and were used independently for the PRO-seq and AID CUT&RUN experiments, respectively.

### Hi-C and Data Analysis.

Hi-C experiments were performed using an Arima-HiC Kit (Arima, A510008) according to the manufacturer's instructions. Sequencing libraries were generated with the NEBNext Ultra II DNA Library Prep Kit (NEB, E7645) according to the manufacturer's guidelines. Libraries were sequenced on a NextSeq 2000 (Illumina) with 150-bp paired-end reads. Hi-C sequencing data were processed using the CPU version of Juicer v1.6 (https://github.com/aidenlab/juicer). Reads were aligned to the modified hg38 reference genome containing the engineered *IGH* allele using the default Juicer alignment workflow. To minimize mapping artifacts arising from the high sequence similarity and repetitive nature of the *IGH* locus, we retained only Hi-C read pairs with a mapping quality (MAPQ) score ≥30 for downstream analysis. This filtering removed a substantial fraction of Hi-C reads from the constant region portion of the *IGH* locus but had little effect on read coverage across most of the genome. The contact maps were visualized in Juicebox.

### Statistical Analysis.

Statistical analyses in Fig. 3*D* and *SI Appendix*, Figs. S1, S3, S4*F*, S5*D*, and S6*A* were performed using a two-tailed unpaired Student's *t* test. Statistical analyses in Figs. 2*B* and 5*C* were performed using a two-sided Pearson correlation test.

## Supplementary Material

Appendix 01 (PDF)

## Data Availability

Sequencing data generated in this study have been deposited in the Gene Expression Omnibus (GEO) under accession number (GSE339325) (63).

## References

[1] Y. Zhang, X. Zhang, H. Q. Dai, H. Hu, F. W. Alt, The role of chromatin loop extrusion in antibody diversification. Nat. Rev. Immunol. **22**, 550–566 (2022). 10.1038/s41577-022-00679-3 PMC9376198 35169260

[2] M. Muramatsu , Class switch recombination and hypermutation require activation-induced cytidine deaminase (AID), a potential RNA editing enzyme. Cell **102**, 553–563 (2000). 10.1016/s0092-8674(00)00078-7 11007474

[3] W. T. Yewdell, K. C. Fernandez, S. M. Downs-Canner, J. Chaudhuri, The molecular logic of immunoglobulin heavy chain class switch recombination. Annu. Rev. Immunol. **44**, 527–551 (2026). 10.1146/annurev-immunol-082323-121803 42045110

[4] R. Casellas , Mutations, kataegis and translocations in B cells: Understanding AID promiscuous activity. Nat. Rev. Immunol. **16**, 164–176 (2016). 10.1038/nri.2016.2 PMC4871114 26898111

[5] J. Ridani, J. Ridani, P. Barbulescu, A. Martin, J. M. D. Noia, "Somatic hypermutation" in Molecular Biology of B Cells, T. Honjo, M. Reth, A. Radbruch, F. Alt, A. Martin, Eds. (Elsevier, 2023), pp. 235–256.

[6] Y. Zhang , The fundamental role of chromatin loop extrusion in physiological V(D)J recombination. Nature **573**, 600–604 (2019). 10.1038/s41586-019-1547-y PMC6867615 31511698

[7] M. Mikhova , A dynamic RNA hub facilitates activation-induced cytidine deaminase recruitment to the immunoglobulin heavy-chain locus. Mol. Cell **86**, 2509–2520.e5 (2026). 10.1016/j.molcel.2026.06.012 PMC13336316 42335900

[8] L. S. Yeap , Sequence-intrinsic mechanisms that target AID mutational outcomes on antibody genes. Cell **163**, 1124–1137 (2015). 10.1016/j.cell.2015.10.042 PMC4751889 26582132

[9] J. Dong , Orientation-specific joining of AID-initiated DNA breaks promotes antibody class switching. Nature **525**, 134–139 (2015). 10.1038/nature14970 PMC4592165 26308889

[10] E. Pinaud , The IgH locus 3′ regulatory region: Pulling the strings from behind. Adv. Immunol. **110**, 27–70 (2011). 10.1016/B978-0-12-387663-8.00002-8 21762815

[11] J. Chaudhuri , Transcription-targeted DNA deamination by the AID antibody diversification enzyme. Nature **422**, 726–730 (2003). 10.1038/nature01574 12692563

[12] E. Pinaud , Localization of the 3′ IgH locus elements that effect long distance regulation of class switch recombination. Immunity **15**, 187–199 (2001). 10.1016/s1074-7613(01)00181-9 11520455

[13] C. Vincent-Fabert , Genomic deletion of the whole IgH 3′ regulatory region (hs3a, hs1, 2, hs3b, and hs4) dramatically affects class switch recombination and Ig secretion to all isotypes. Blood **116**, 1895–1898 (2010). 10.1182/blood-2010-01-264689 20538806

[14] X. Zhang , Fundamental roles of chromatin loop extrusion in antibody class switching. Nature **575**, 385–389 (2019). 10.1038/s41586-019-1723-0 PMC6856444 31666703

[15] Y. Fukita, H. Jacobs, K. Rajewsky, Somatic hypermutation in the heavy chain locus correlates with transcription. Immunity **9**, 105–114 (1998). 10.1016/s1074-7613(00)80592-0 9697840

[16] A. Tarsalainen , Ig enhancers increase RNA polymerase II stalling at somatic hypermutation target sequences. J. Immunol. **208**, 143–154 (2022). 10.4049/jimmunol.2100923 PMC8702490 34862258

[17] J. M. Buerstedde, J. Alinikula, H. Arakawa, J. J. McDonald, D. G. Schatz, Targeting of somatic hypermutation by immunoglobulin enhancer and enhancer-like sequences. PLoS Biol. **12**, e1001831 (2014). 10.1371/journal.pbio.1001831 PMC3972084 24691034

[18] P. Rouaud , The IgH 3′ regulatory region controls somatic hypermutation in germinal center B cells. J. Exp. Med. **210**, 1501–1507 (2013). 10.1084/jem.20130072 PMC3727322 23825188

[19] A. Saintamand , Deciphering the importance of the palindromic architecture of the immunoglobulin heavy-chain 3′ regulatory region. Nat. Commun. **7**, 10730 (2016). 10.1038/ncomms10730 PMC4757795 26883548

[20] C. L. Morvan, E. Pinaud, C. Decourt, A. Cuvillier, M. Cogne, The immunoglobulin heavy-chain locus hs3b and hs4 3′ enhancers are dispensable for VDJ assembly and somatic hypermutation. Blood **102**, 1421–1427 (2003). 10.1182/blood-2002-12-3827 12714490

[21] U. Storb, Why does somatic hypermutation by AID require transcription of its target genes? Adv. Immunol. **122**, 253–277 (2014). 10.1016/B978-0-12-800267-4.00007-9 24507160

[22] F. Senigl , Topologically associated domains delineate susceptibility to somatic hypermutation. Cell Rep. **29**, 3902–3915.e3908 (2019). 10.1016/j.celrep.2019.11.039 PMC6980758 31851922

[23] U. E. Schoeberl , Regulation of somatic hypermutation by higher-order chromatin structure. Mol. Cell **85**, 2701–2717.e9 (2025). 10.1016/j.molcel.2025.06.003 40580953

[24] J. E. Sale, M. S. Neuberger, TdT-accessible breaks are scattered over the immunoglobulin V domain in a constitutively hypermutating B cell line. Immunity **9**, 859–869 (1998). 10.1016/s1074-7613(00)80651-2 9881976

[25] F. N. Papavasiliou, D. G. Schatz, Cell-cycle-regulated DNA doublestrand breaks in somatic hypermutation of immunoglobulin genes. Nature **408**, 216–220 (2000). 10.1038/35041599 11089977

[26] R. K. Dinesh , Transcription factor binding at Ig enhancers is linked to somatic hypermutation targeting. Eur. J. Immunol. **50**, 380–395 (2020). 10.1002/eji.201948357 PMC7202714 31821534

[27] U. E. Schoeberl , Somatic hypermutation patterns in immunoglobulin variable regions are established independently of the local transcriptional landscape. eLife **14**, RP106566 (2025).

[28] M. Bemark, M. S. Neuberger, The c-MYC allele that is translocated into the IgH locus undergoes constitutive hypermutation in a Burkitt's lymphoma line. Oncogene **19**, 3404–3410 (2000). 10.1038/sj.onc.1203686 10918597

[29] M. Gostissa , Long-range oncogenic activation of Igh-c-myc translocations by the Igh 3′ regulatory region. Nature **462**, 803–807 (2009). 10.1038/nature08633 PMC2802177 20010689

[30] M. Wang, Z. Yang, C. Rada, M. S. Neuberger, AID upmutants isolated using a high-throughput screen highlight the immunity/cancer balance limiting DNA deaminase activity. Nat. Struct. Mol. Biol. **16**, 769–776 (2009). 10.1038/nsmb.1623 PMC4851237 19543289

[31] L. Wu , HMCES protects immunoglobulin genes specifically from deletions during somatic hypermutation. Genes Dev. **36**, 433–450 (2022). 10.1101/gad.349438.122 PMC9067407 35450882

[32] L. Wu , Transcription elongation factor ELOF1 is required for efficient somatic hypermutation and class switch recombination. Mol. Cell **85**, 1296–1310.e7 (2025). 10.1016/j.molcel.2025.02.007 PMC11972161 40049160

[33] P. Dai , Transcription-coupled AID deamination damage depends on ELOF1-associated RNA polymerase II. Mol. Cell **85**, 1280–1295.e9 (2025). 10.1016/j.molcel.2025.02.006 40049162

[34] H. Chen , BCR selection and affinity maturation in Peyer's patch germinal centres. Nature **582**, 421–425 (2020). 10.1038/s41586-020-2262-4 PMC7478071 32499646

[35] S. Jain, Z. Ba, Y. Zhang, H. Q. Dai, F. W. Alt, CTCF-binding elements mediate accessibility of RAG substrates during chromatin scanning. Cell **174**, 102–116.e114 (2018). 10.1016/j.cell.2018.04.035 PMC6026039 29804837

[36] P. D′Addabbo, M. Scascitelli, V. Giambra, M. Rocchi, D. Frezza, Position and sequence conservation in Amniota of polymorphic enhancer HS1.2 within the palindrome of IgH 3′Regulatory Region. BMC Evol. Biol. **11**, 71–83 (2011). 10.1186/1471-2148-11-71 PMC3068965 21406099

[37] B. Laffleur , AID-induced remodeling of immunoglobulin genes and B cell fate. Oncotarget **5**, 1118–1131 (2013). 10.18632/oncotarget.1546 PMC4012742 24851241

[38] G. Sharbeen, C. W. Yee, A. L. Smith, C. J. Jolly, Ectopic restriction of DNA repair reveals that UNG2 excises AID-induced uracils predominantly or exclusively during G1 phase. J. Exp. Med. **209**, 965–974 (2012). 10.1084/jem.20112379 PMC3348097 22529268

[39] Q. Le, N. Maizels, Cell cycle regulates nuclear stability of AID and determines the cellular response to AID. PLoS Genet. **11**, e1005411 (2015). 10.1371/journal.pgen.1005411 PMC4565580 26355458

[40] Q. Wang , The cell cycle restricts activation-induced cytidine deaminase activity to early G1. J. Exp. Med. **214**, 49–58 (2017). 10.1084/jem.20161649 PMC5206505 27998928

[41] J. Pae , Transient silencing of hypermutation preserves B cell affinity during clonal bursting. Nature **641**, 486–494 (2025). 10.1038/s41586-025-08687-8 PMC12058519 40108454

[42] J. Merkenschlager , Regulated somatic hypermutation enhances antibody affinity maturation. Nature **641**, 495–502 (2025). 10.1038/s41586-025-08728-2 PMC12058521 40108475

[43] A. Faili , AID-dependent somatic hypermutation occurs as a DNA single-strand event in the BL2 cell line. Nat. Immunol. **3**, 815–821 (2002). 10.1038/ni826 12145648

[44] S. S. P. Rao , Cohesin loss eliminates all loop domains. Cell **171**, 305–320.e324 (2017). 10.1016/j.cell.2017.09.026 PMC5846482 28985562

[45] M. Gabriele , Dynamics of CTCF- and cohesin-mediated chromatin looping revealed by live-cell imaging. Science **376**, 496–501 (2022). 10.1126/science.abn6583 PMC9069445 35420890

[46] Z. Ba , CTCF orchestrates long-range cohesin-driven V(D)J recombinational scanning. Nature **586**, 305–310 (2020). 10.1038/s41586-020-2578-0 PMC7554077 32717742

[47] H. Q. Dai , Loop extrusion mediates physiological Igh locus contraction for RAG scanning. Nature **590**, 338–343 (2021). 10.1038/s41586-020-03121-7 PMC9037962 33442057

[48] B. Nabet , Rapid and direct control of target protein levels with VHL-recruiting dTAG molecules. Nat. Commun. **11**, 4687 (2020). 10.1038/s41467-020-18377-w PMC7501296 32948771

[49] Y. Cheng , A 3D genome atlas of human tonsil and the role of loop extrusion in B cell somatic hypermutation. Science **393**, eadw4243 (2026). 10.1126/science.adw4243 PMC13464094 42490500

[50] G. D. Victora, M. C. Nussenzweig, Germinal centers. Annu. Rev. Immunol. **40**, 413–442 (2022). 10.1146/annurev-immunol-120419-022408 35113731

[51] M. C. Lauring , Co-option of retrotransposons promotes antibody diversification. Cell **189**, 1–21 (2026). 10.1016/j.cell.2026.07.021 PMC13656868 42546688

[52] S. P. Methot , A licensing step links AID to transcription elongation for mutagenesis in B cells. Nat. Commun. **9**, 1248 (2018). 10.1038/s41467-018-03387-6 PMC5871760 29593215

[53] A. Peters, U. Storb, Somatic hypermutation of immunoglobulin genes is linked to transcription initiation. Immunity **4**, 57–65 (1996). 10.1016/s1074-7613(00)80298-8 8574852

[54] R. Pavri , Activation-induced cytidine deaminase targets DNA at sites of RNA polymerase II stalling by interaction with Spt5. Cell **143**, 122–133 (2010). 10.1016/j.cell.2010.09.017 PMC2993080 20887897

[55] X. Zhang, H. S. Yoon, A. M. Chapdelaine-Williams, N. Kyritsis, F. W. Alt, Physiological role of the 3′IgH CBEs super-anchor in antibody class switching. Proc. Natl. Acad. Sci. U.S.A. **118**, e2024392118 (2021). 10.1073/pnas.2024392118 PMC7826415 33441485

[56] A. M. Patenaude , Active nuclear import and cytoplasmic retention of activation-induced deaminase. Nat. Struct. Mol. Biol. **16**, 517–527 (2009). 10.1038/nsmb.1598 19412186

[57] Q. Le, N. Maizels, Activation-induced deaminase (AID) localizes to the nucleus in brief pulses. PLoS Genet. **15**, e1007968 (2019). 10.1371/journal.pgen.1007968 PMC6411215 30811383

[58] E. Pefanis , RNA exosome-regulated long non-coding RNA transcription controls super-enhancer activity. Cell **161**, 774–789 (2015). 10.1016/j.cell.2015.04.034 PMC4428671 25957685

[59] B. R. Sabari , Coactivator condensation at super-enhancers links phase separation and gene control. Science **361**, eaar3958 (2018). 10.1126/science.aar3958 PMC6092193 29930091

[60] A. S. Weintraub , YY1 is a structural regulator of enhancer-promoter loops. Cell **171**, 1573–1588.e1528 (2017). 10.1016/j.cell.2017.11.008 PMC5785279 29224777

[61] J. Hu , Detecting DNA double-stranded breaks in mammalian genomes by linear amplification-mediated high-throughput genome-wide translocation sequencing. Nat. Protoc. **11**, 853–871 (2016). 10.1038/nprot.2016.043 PMC4895203 27031497

[62] D. B. Mahat , Base-pair-resolution genome-wide mapping of active RNA polymerases using precision nuclear run-on (PRO-seq). Nat. Protoc. **11**, 1455–1476 (2016). 10.1038/nprot.2016.086 PMC5502525 27442863

[63] F. W. Alt, X. Chen, Y. Guan, Data from "Human antibody heavy chain variable region exon interacts with 3′ regulatory region to form a stable somatic hypermutation center." Gene Expression Omnibus (GEO), NCBI. https://www.ncbi.nlm.nih.gov/geo/query/acc.cgi?acc=GSE339325. Deposited 11 September 2026. 10.1073/pnas.2625638123 PMC13643152 42804635

